# Is the List of Ingredients a Source of Nutrition and Health Information in Food Labeling? A Scoping Review

**DOI:** 10.3390/nu15214513

**Published:** 2023-10-25

**Authors:** Mariana V. S. Kraemer, Ana Carolina Fernandes, Maria Cecília C. Chaddad, Paula L. Uggioni, Greyce L. Bernardo, Rossana P. C. Proença

**Affiliations:** 1 Nutrition in Foodservice Research Centre, Nutrition Postgraduate Program, Federal University of Santa Catarina, Campus Universitário, Centro de Ciências da Saúde, Bloco C–2º andar, Trindade, Florianópolis 88040-900, SC, Brazil; marianavskraemer@gmail.com (M.V.S.K.); ana.fernandes@ufsc.br (A.C.F.); paula.uggioni@ufsc.br (P.L.U.); greyce.bernardo@ufsc.br (G.L.B.); 2 Movimento Põe no Rótulo, R. Ministro Godói, 969, Perdizes, São Paulo 05015-000, SP, Brazil; cecilia@ceciliacury.adv.br

**Keywords:** nutrition labelling, labelling regulations, nutrition policies, food regulation, Codex Alimentarius, scoping review

## Abstract

Nutrition labelling is any description intended to inform consumers about the nutritional properties of a food product and has focused primarily on nutrients. However, literature has shown that the nutritional quality of packaged foods is not limited to the amount of nutrients, considering that individuals do not consume only nutrients separately, but rather the entire food matrix. Therefore, to analyze the nutritional quality of a packaged food, it is necessary to read its ingredients. This scoping review aims to discuss (1) the list of ingredients as a source of health and nutrition information in food labelling; (2) opportunities to improve the nutrition labeling policies around the world. The study was carried out through a systematic search on Codex Alimentarius meeting reports. Results show that the list of ingredients is used as a source of nutritional and health information on food labelling; however, this label item is not considered in the regulatory field as a nutrition labelling requirement. It is suggested that nutrition labelling be discussed as a tool for food choices in the context of public health from a broader, consistent, convergent perspective, considering the list of ingredients as an item of nutrition labelling requirement to be included in public policies around the world.

## 1. Introduction

Food labeling encompasses any information available on packaged foods, be it in written, printed, lithographed, embossed, impressed, or attached form [1]. In the current context of food consumption and market relationships, food labels serve as one of the most important and direct means of communicating information to consumers. Labels must contain clear and reliable information on the identity and content of the product as well as on how to handle, prepare, and consume it safely [2]. 

Each country has the autonomy to establish its own labeling regulations, defining what type of information is mandatory and how it should be presented. International standards recommend the adoption of the list of ingredients as a mandatory item on food labels [1]. It is the only information that allows consumers to identify which substances are present in a food product. As ingredients should be listed in descending order of quantity, consumers can use the list of ingredients to gain an overview of the proportion of each ingredient. However, despite the importance of the list of ingredients as a source of information on health and nutrition [3] to assist consumers in making more informed food choices [4], this label item is not treated in the regulatory field as a nutrition labeling requirement.

Nutrition labeling includes any description intended to inform consumers about the nutritional properties of a food product. Its presentation is divided into two elements: nutrient declaration and supplementary nutrition information [4]. The nutrient declaration is presented in the nutrition facts panel and consists of a standardized table or list of nutrients contained in a food product. Supplementary nutrition information is intended to increase the consumer’s understanding of the nutritional value of their food and to assist in interpreting the nutrient declaration. An example of supplementary nutrition information is a nutrition claim, which is defined as any statement that affirms, suggests, or implies that a food has specific nutritional properties, including but not limited to references to energy value and contents of proteins, fats, carbohydrates, vitamins, and minerals [4]. 

It can be understood that nutrition labeling, in its basic form, has focused primarily on nutrients, although some nutrition claims may require the analysis of the list of ingredients for implementation. In view of the increasing application of food technologies and the growing use of non-food ingredients and substances extracted or derived from foods [5], the list of ingredients seems to have acquired an expanded function, serving as a source of health and nutrition information. This study aimed to discuss (1) the role of the list of ingredients as a source of health and nutrition information in food labeling, and (2) opportunities to improve the nutrition labeling policies around the world.

## 2. Materials and Methods

This scoping review was conducted on the basis of recommendations of the Joanna Briggs Institute (JBI) [6] and the checklist of Preferred Reporting Items for Systematic Reviews and Meta-Analyses Extension for Scoping Reviews (PRISMA-ScR) [7]. According to JBI, scoping reviews are conducted for several reasons and unlike other reviews that tend to address relatively precise questions, such as systematic reviews, “scoping reviews can be used to map the key concepts that underpin a field of research, as well as to clarify working definitions, and/or the conceptual boundaries of a topic” [6].

Initially, as recommended by JBI, we developed a protocol containing the guiding question, PCC (Population, Concept, and Context) framework, inclusion criteria for official documents, and an outline of contents covered in each section of the manuscript (Supplementary Material S1). The aim of the PCC framework is to be a guide to construct a clear and meaningful title for a scoping review [6].

The search was carried out on the Codex Alimentarius website and focused on identifying official documents issued by the World Health Organization (WHO) and the Food and Agriculture Organization of the United Nations (FAO) and meeting reports of the Codex Committee on Food Labelling (CCFL) since its creation, in 1965, to the present day [8]. This search was designed to retrieve documents that would allow us to assess under which premises the list of ingredients is discussed as a mandatory requirement on food labels and examine its role as a source of health and nutrition information in the preparation of recommendations on food labeling. For this, we have searched for the uniterms “ingredient”, “ingredients”, “list of ingredients”, “health”, and “nutrition” in the Codex Alimentarius documents. Additional searches were carried out on government websites and websites of national regulatory agencies.

For data analysis, a content analysis was carried out on all documents found during the search, in order to screen the discussion about the list of ingredients. As inclusion criteria, all meeting reports of the Codex Committee on Food Labelling (1965–2023) were included. 

## 3. Results and Discussion

### 3.1. The List of Ingredients as Discussed by the Codex Committee on Food Labelling: Historical Milestones from a Health and Nutrition Perspective

The Codex Alimentarius was created by FAO in 1961. In 1963, it became a joint FAO/WHO program aimed at developing a collection of standards, guidelines, and principles for foods through committee meetings with government representatives and experts [9]. The main objectives of the program are to protect consumer health, ensure fair trade practices in the food sector, and promote the harmonization of food standards [10].

In 1965, the Codex Alimentarius Commission recommended the creation of standard guidelines on food labeling, which resulted in the establishment of the Codex Committee on Food Labelling. The Committee, composed mostly of government and food industry representatives from different countries, meets periodically to discuss and improve the General Standard for the Labelling of Prepackaged Foods, which was released in 1985, amended in 1991, 1999, 2001, 2003, 2005, 2008, and 2010, and thoroughly revised in 2018.

The discussions that served as a basis for recommendations and guidelines on food labeling of the General Standard for the Labelling of Prepackaged Foods can be found in the minutes of the Codex Committee on Food Labelling. The first meeting took place in 1965. Since then, 47 meetings have been held to discuss food labeling [10]. Sometimes, subcommittees are created to advise on specific issues that require in-depth analysis. Discussions also serve for the development of recommendation guidelines, rather than standards, such as the Guidelines on Nutrition Labelling and the Guidelines for Use of Nutrition and Health Claims. Such recommendations are based on the principle that food labels must not contain information that is false, misleading, or deceptive or that might suggest an erroneous relationship between products [1]. Additionally, considering that food labeling is a cross-cutting subject in some Codex Alimentarius Committees, such as Codex Committee on Nutrition and Foods for Special Dietary Uses (CCNFSDU), Codex Committee on Food Additives (CCFA), and Commodity Committees, occasionally, there is a joint work between committees.

By analyzing meeting reports since the Committee’s creation in the 1960s, although one of the main objectives of the Codex Alimentarius is to protect consumer health, the development of labeling standards for packaged foods has also had a commercial focus, associated with the guarantee of free and fair trade within and between countries. The lists of participants suggest that the food commodity and technology sectors had a strong influence on debates and documents approved by the Codex Alimentarius.

Although the importance of food labels to consumers is acknowledged in discussions leading to the adoption of the General Standard for the Labelling of Prepackaged Foods, it was found that the final version of the document does not contain the term “health” [1]. That is, there are no explicit recommendations relating food labeling to health aspects in the major international reference on the topic. Two occurrences of the word “nutrition” were found, one within the concept of claims (nutrition claims) and the other related to food additives (specifying that they should have no nutritional value). It can be stated, therefore, that these recommendations aim to promote global harmonization of food labeling regulations and ensure consumers’ right to clear and reliable information on food contents and manufacture [1] but without considering health and nutrition as a focus or underlying principle.

From the first to the last version of the General Standard for the Labelling of Prepackaged Foods, the list of ingredients is defined as a mandatory requirement, except for foods composed of a single ingredient [1]. For all other foods, the list of ingredients must disclose all ingredients in descending order of quantity according to the amounts added at the time of manufacture. Other important requirements for the declaration of the list of ingredients are as follows:

- Where an ingredient is itself the product of two or more ingredients, it is called a compound ingredient. The ingredients of a compound ingredient shall be declared whenever the compound ingredient constitutes more than 5% of the food;
- Some ingredients shall always be declared in the list of ingredients because of their potential to cause hypersensitivity (cereals containing gluten, crustaceans, eggs, fish, milk, peanuts, soybean, nuts, and sulfites in concentrations greater than 10 mg/kg);
- Some ingredients can be declared by the name of the food class to which it belongs, such as “sugar” for all types of sucrose and “cheese” for all types of cheese;
- Food additives shall be declared by the name of the substance or by their identification in the International Numbering System, always preceded by the functional class.

In a section entitled Additional Mandatory Requirements, the Committee recommends the quantitative declaration of ingredients. This topic began to be discussed with greater emphasis from the early 2000s onward. After ample debate and oftentimes divergence among committee members, it was established that the percentage of an ingredient in relation to the total weight or volume of product should be disclosed when (i) its presence is emphasized on the label through words, pictures, or graphics, or (ii) it is essential to characterize the food, and the omission of the quantity may deceive consumers [1].

**Figure 1:**
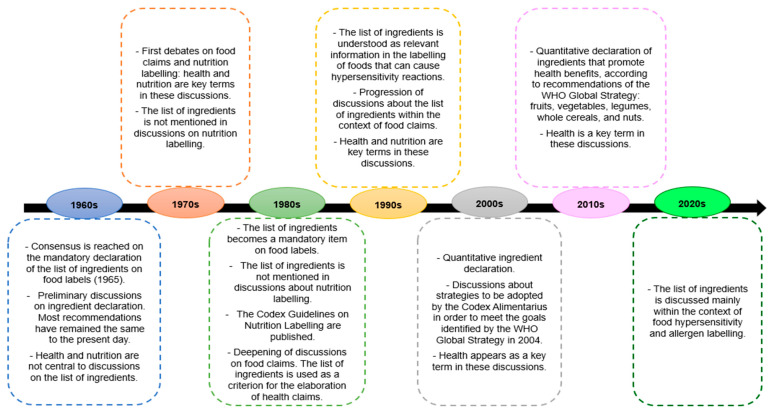
Timeline (1960–2023) of milestones in discussions held by the Codex Committee on Food Labelling on the role of the list of ingredients as a source of health and nutrition information on food labels. Sources: [11,12,13,14,15,16,17,18,19,20,21,22,23,24,25,26,27,28,29,30,31,32,33,34,35,36,37,38,39,40,41,42,43,44,45,46,47,48,49,50,51,52,53,54,55,56,57].

Figure 1 illustrates a timeline summarizing the reports in which there were discussions held by the Codex Committee on Food Labelling from 1965 to 2023 about the relevance of the list of ingredients, with a special focus on health and nutrition [8].

Since 1965, there have been several discussions within the framework of the Codex Alimentarius on the matter of what and how to present information on food labels. During the 1960s, the need to disclose the complete list of ingredients to consumers was a recurrent theme. Such debates were punctuated by several arguments both against and in favor of the full declaration of the list of ingredients. Some participants pointed out that declaring all ingredients contained in a food product without disclosing quantities or proportions would be inaccurate, imprecise, and of little use to consumers. In the view of other representatives, consumers might not understand the meaning of substances declared in the list of ingredients. These arguments were often refuted, and points raised in favor of the full declaration of the list of ingredients prevailed, such as the right of consumers to receive correct and reliable information, the fact that some religious and cultural norms do not allow the consumption of certain foods, and the existence of food-related health conditions such as food allergies and hypersensitivities [11,12,13,14].

During the 1970s, little reference was made to the list of ingredients in the context of health and nutrition. Throughout this decade, the Committee produced two guidelines, the Guidelines of Food Claims and the Guidelines of Nutrition Labelling. It became evident that the terms “health” and “nutrition” were gaining prominence in the minutes of committee meetings. These terms were often brought up during conversations about food claims and nutrition labeling, but the list of ingredients was never mentioned within these contexts [15,16,17,18,19,20,21,22,23,24]. Nevertheless, although the list of ingredients was not explicitly considered and discussed as a source of nutrition information, it often permeated discussions on food claims and nutrition labeling. The following excerpt can be found in the minutes of the 15th meeting of the Codex Committee on Food Labelling in 1980:

“[…] certain long-chain fatty acids, when exposed to high temperatures, would be transformed into substances which were detrimental to the health of the consumer. […] the label should contain reference to the source of fat, since not all types of fat were acceptable to all population groups in their countries for religious reasons. It was agreed that this matter could be considered in conjunction with the Revision of the General Standard (List of Ingredients).”

[25] (p. 12)

As stated in the minutes of the 15th meeting, members of the Australian delegation explained their position on the declaration of nutrients in food labels by giving the following statement:

“[…] allow for the declaration of energy values without triggering the need for full nutrition labelling since energy values, taken in conjunction with the list of ingredients, would provide consumers in many countries with useful information.”

[25] (p. 13)

Thus, as exemplified by these two excerpts, the list of ingredients is emphasized as relevant information complementary to nutritional, cultural, and health aspects. From the perspective of the delegation of Australia, the list of ingredients is an important item that seems to provide more relevant information to consumers than a quantitative declaration of nutrients, considering that the delegation did not state a full nutrition labeling as necessary information. In this regard, discussions of the Committee seem to endorse the view that information on the source of nutrients may be as important as information on the amount of nutrients contained in foods. 

During the 1980s and 1990s, debates on guidelines for nutrition labeling and food claims progressed and deepened [25,26,27,28,29,30,31,32,33,34,35,36,37]. Other important documents were published, mainly in the 1990s, such as the Guidelines for the Production, Processing, Labelling and Marketing of Organically Produced Foods and the Recommendations for the Labelling of Foods that Can Cause Hypersensitivity. These recommendations encompass discussions on food ingredients, mainly in the context of identification of organic foods and potential allergens, as well as a criterion for the elaboration of some nutrition claims. On the topic of negative claims, that is, statements indicating the absence of an ingredient or nutrient (e.g., additive free), the Committee raised concerns that claims might contradict information declared in the list of ingredients, creating an erroneous impression of food products and their uses. The following excerpt about the Guidelines of Food Claims was taken from the report of the 18th session of the Codex Committee on Food Labelling in 1985:

“[…] to prohibit negative claims on the basis that they cast doubt not only on comparable products and the ingredients contained therein but also on the validity of compulsory lists of ingredients and food technology in general. Such declarations tend to emphasize qualities which are often only marginal and may therefore give a completely wrong impression of the food and its use.”

[28] (p. 125)

In the 2000s, in line with trends of previous decades, the list of ingredients was invoked in discussions on nutrition labeling and understood as an additional piece of information relevant to health and nutrition. The minutes of the 28th session of the Codex Committee, held in 2000, contain the following statements about the declaration of protein in nutrition labeling:

“The Committee noted a proposal to include a reference to the source of protein. The Committee however recalled that the purpose of the Guidelines was to provide information on the nutrient contents while the General Standard for the Labelling of Prepackaged Foods provided the relevant information on the source of nutrients through the declaration of ingredients, which was always included in the labelling.”

[38] (p. 7)

From 2000 to 2011, there were relevant debates on the role of the list of ingredients [38,39,40,41,42,43,44,45,46,47,48,49]. Quantitative declaration of ingredients was on the agenda of several meetings and regarded as a tool to help consumers avoid misinterpretation of food labels and make more informed food choices, especially those related to health issues and the nutritional quality of food. One proposal was to declare in the list of ingredients the percentage of each ingredient in relation to the total weight of the food. Several arguments were raised, both against and in favor of the quantitative declaration of ingredients. Contrary arguments included concerns about the violation of manufacturers’ intellectual property rights, the possibility of discouraging innovations in product development, and the creation of barriers to free trade. Favorable arguments focused on the potential of the quantitative declaration of ingredients to ensure fair practices in the food market and protect consumer health. Other points merited discussion, as exemplified in the following excerpts extracted from the minutes of the 31st and 32nd sessions of the Committee in 2003 and 2004:

“[…] QUID (quantitative declaration of ingredients) would be helpful for consumer’s choice and especially in view of the increased interest in nutritional information.”

[41] (p. 12)

“[…] this would help consumers to make an informed choice and facilitate their understanding of nutritional information.”

[42] (p. 11)

“[…] FAO/WHO Expert Report No. 916 identified several foods (commonly used as ingredients in processed foods) which have effects, distinct from known nutrient effects, on major disease risks and therefore, national authorities should be permitted to require QUID for these ingredients regardless of whether claims are made.”

[42] (p. 11)

It can be seen that the list of ingredients is regarded by the Committee as nutrition and health information. The WHO report mentioned in the last excerpt reiterates the role of foods, rather than nutrients, in the prevention of diseases. Furthermore, regardless of nutrition claims or nutrient amounts, the declaration of all ingredients and/or substances added to a food product is relevant from a health and nutrition perspective.

As of 2006, a topic permeating the meetings of the Codex Alimentarius was the WHO Global Strategy on Diet, Physical Activity, and Health. This initiative, launched in 2004, focused on preventing and controlling the development of chronic non-communicable diseases [58]. The Global Strategy was discussed within the framework of the Codex Alimentarius on Food Labelling with the aim of developing strategies on food labeling to attain the proposed goals [44,45,46,47,48,49]. The list of ingredients was a recurrent theme and often considered a key topic in these discussions. WHO representatives noted that:

“[…] information on the nutrient content of a prepackaged food was as necessary as information on the ingredients in enabling a consumer to make an informed choice of foods.”

[45] (p. 4)

Other statements reinforced that the nutritional quality of a food does not depend solely on its nutrient content. During the 35th session of the Committee in 2007, WHO members pointed out:

“The health benefits from fruit, vegetables, whole grains and legumes are related not only to the nutrients but also to many other substances present in these foods and in some cases to the matrix provided by the intact food, and as such are not covered by Codex texts pertaining to nutrition labelling or claims.”

[45] (p. 15)

It is noteworthy that WHO representatives, as well as other delegations participating in Codex Alimentarius Committees, see the inclusion of quantitative declaration of ingredients as key to health promotion, especially for ingredients identified in the Global Strategy, namely fruits, vegetables, legumes, whole grains, sugars, and salt. Discussions have shed light on the role of the list of ingredients in providing relevant nutrition and health information to consumers. However, there has been no progress with respect to recommendations on this topic. To date, quantitative declaration of ingredients has not been included as mandatory and/or voluntary information in the General Standard for the Labelling of Prepackaged Foods.

After analyzing discussions on nutrition labeling held by the Committee, it became evident the widespread notion that nutrition information is synonymous with quantitative nutrient declaration. However, it is currently understood that information on the quantity of nutrients does not sufficiently encompass all nutrition and health aspects that should be informed to the consumer. Discussions on the list of ingredients frequently delve into commercial and fair-trade issues of food production and tacitly refer to it as complementary information that supports health and nutrition choices and, particularly, the elaboration of food claims and nutrition labels. 

Whereas the Committee that defines global standards for food products and serves as a basis for labeling regulations worldwide [59] does not officially recognize the list of ingredients as nutrition information on food labels, it was observed that the national regulations on nutrition labeling do not consider the list of ingredients as a component of nutrition labeling [60,61,62]. This means that the ingredients list is probably not discussed by the government agencies as a nutrition and health tool in food labeling. An example of a potential consequence of this scenario happened in Brazil. In 2020, Brazil approved a new regulation for nutrition labeling, making a Front of Pack (FoP) model mandatory for products with excessive amounts of added sugar, sodium, and saturated fat. During the regulatory process, academia and advocacy organizations suggested to the Brazilian Regulatory Agency (ANVISA) the inclusion of sweeteners as an item on the FoP model. However, ANVISA did not accept this suggestion, based on the fact that sweeteners are not in the scope of nutrition labeling, because they are only declared in the ingredients list. The following excerpt can be found in the document published by ANVISA [63]:

“Regarding the proposals for the inclusion of sweeteners [as a FofP item], as already clarified in the Preliminary Report on Nutritional Labeling, these substances are ingredients intentionally added to foods for technological purposes. Therefore, they are not classified as nutrients. (…) Thus, concerns related to the safety of using these substances (...) are not in the scope of the regulatory process on nutrition labelling and do not justify the inclusion of information about the presence of this substance in the Front of Pack Nutrition Labelling.”.

[63] (pp. 90, 91)

This issue has become even more relevant with the recent WHO Guidelines on non-sugar sweeteners (NSS), which recommends not to use NSS to control body weight or to reduce the risk of noncommunicable diseases (NCDs). In the document, it is stated that the Guideline “can be considered by policy-makers and programme managers when discussing possible measures, including nutrition labelling” [64] (p. 24). However, how can this recommendation be effectively implemented in the regulatory field if sweeteners (and all food additives) are declared only in the list of ingredients, which is not considered an item of nutrition labeling?

This scenario may have additional consequences for consumers. Using the same example of the Brazilian new regulation on nutrition labeling, the declaration of added sugars was also approved as mandatory on the nutrition information panel. If the list of ingredients was a nutrition labeling item, it could also have been updated to declare the sources of added sugars grouped in the list, thereby assisting consumers in making more informed choices. Nevertheless, improvements to the declaration of the content of the ingredients list were not made together with nutrition labeling improvements.

Review studies have shown that the use of the nutrition facts panel seems to positively influence food quality [65,66], although the influence of the list of ingredients remains to be elucidated. In a review study assessing the relationship between the use of food label information and food consumption, the authors highlighted the need to analyze the impact of other nutrition information items, such as the list of ingredients, on diet quality improvement [3]. In this sense, such as in Brazil, models for improving the understanding and readability of nutrition labeling are studied around the world [67]. However, little is known about improving the list of ingredients declaration as well as the impact on consumers’ understanding of food labeling. It may happen because the list of ingredients does not present nutrients and, therefore, is not part of the scope of nutrition labeling.

Despite the arguments presented above on the relevance of the list of ingredients as nutrition information on food labels, consumer rights, concerns with fair trade, and minimization of food fraud remain as premises in the guidelines of the Codex Alimentarius for Food Labelling [1]. The legitimacy and importance of such issues in the regulatory field are undisputed; however, the list of ingredients is discussed only under these topics. Scientific advances in food and nutrition have shown the need to rethink and debate the role of the list of ingredients, and, particularly, the information contained in it, from a more comprehensive perspective of health and nutrition.

### 3.2. Nutrition Labeling: Do Only Nutrients Matter for Health?

In the global regulatory framework as well as in labeling standards and recommendations, nutrition information is considered a synonym of nutrient content. Similarly, in defining healthy eating, WHO centralizes recommendations on nutrient contents. Although one of the recommendations is to increase the intake of fruits, vegetables, nuts, and whole grains, other guidelines are based on which nutrients should be avoided in order to attain a healthy diet [68].

Other documents published by WHO also established guidelines focused on the nutrient content of packaged foods. The technical report Diet, Nutrition, and the Prevention of Chronic Diseases (2020) determined nutrient intake targets for the population for the prevention of obesity and chronic non-communicable diseases. This WHO report established maximum intake levels of critical nutrients, serving as a criterion for the development of public policies around the world [69]. Overall, it is possible to observe that nutritional analysis of foods is focused on nutrient content.

It is, however, understood that this is not the only possible approach. Scientific literature has shown that the nutritional quality of packaged foods is not limited to the amount of nutrients. Foods are complex combinations of nutrients and other compounds that act synergistically with each other, other foods, and, above all, the human organism [70]. In this context, individuals do not consume only the energy value, macronutrients, or micronutrients contained in foods, but rather the entire food matrix. This food matrix plays an important role in the release and bioavailability of various nutrients, as these nutrients may interact with other food components in the intestine, binding to macromolecules and forming chemical complexes and colloidal structures that reduce or improve bioavailability [71]. Thus, the relationship between food nutritional quality and potential health consequences is complex, requiring more than just the analysis of nutrient content for a thorough understanding [72].

A previous study questioned a public policy on food and nutrition for the reduction of obesity that focused essentially on energy restriction. The authors highlighted that the energy intake approach reduces food to a single aspect, which, in most cases, is not the most important for health promotion. They reinforced the importance of focusing efforts on healthy food and eating patterns to reduce obesity, given that people eat food, not calories. Furthermore, the authors underscored that, to analyze the nutritional quality of a packaged food, it is necessary to read the list of ingredients to obtain information on nutrient sources [73].

In this sense, it is worth noting that it is challenging for consumers to analyze the nutritional quality of a packaged food. Researchers in food and nutrition science and public health policy makers do not state a consensus about the theme. A few criteria have been formulated, such as the nutrient profiling model [74] and processed food classification methods [75]. However, it remains challenging to make a clearer communication towards the concept and criteria to establish what a healthy food is [76]. 

In recent years, dietary guidelines from various countries have shifted toward a more comprehensive perspective on the nutritional quality of foods, no longer focusing solely on nutrients. The Dietary Guidelines for the Brazilian Population is an example of such an approach. The document, published in 2014, provides the country’s official recommendations for adequate and healthy eating with an approach focused on the degree of food processing, production method, and meal composition. Following the NOVA classification [77,78], the guideline recommends that foods with a high degree of processing should be avoided, giving preference to fresh and minimally processed foods. Furthermore, it underscores that, to identify ultraprocessed foods, it is essential to analyze their ingredients [79].

Other dietary guidelines also adopt less nutrient-centric approaches to food. Countries such as Canada, Australia, United States of America, and members of the European Union developed food guides that reinforce the importance of food composition, dietary patterns, and variety of foods for a healthy diet [80,81,82,83]. It is noteworthy that Canada’s Food Guide argues that the list of ingredients should be consulted by the population to base their food choices. According to the document, by reading the list of ingredients, it is possible to know which ingredients are in greater or lesser proportion and identify ingredients to be avoided (such as allergens) [80].

It is thus considered that the nutritional characteristics of a food are more comprehensive than solely the content of energy, macronutrients, and micronutrients. The sources of these nutrients are fundamental to analyze the nutritional quality of a food more accurately and comprehensively from a health and nutrition point of view. 

Considering the high global consumption of packaged foods, it is therefore understood that the list of ingredients can play a central role in the analysis of the nutritional quality of foods, as it provides consumers with more information, thereby allowing informed choices.

### 3.3. Limitations

This was a scoping review, carried out according to JBI Scoping Review Methodology. However, the study has limitations. It was not included the discussions of other Codex Alimentarius Committees, such as Codex Committee on Nutrition and Foods for Special Dietary Uses (CCNFSDU), Codex Committee on Food Additives (CCFA) and Commodity Committees, concerning list of ingredients. However, discussing food labeling, nutrition labeling, and list of ingredients is not the main purpose of these Committees, but of the Codex Committee on Food Labelling (CCFL). Consequently, discussions and decisions around these themes are most likely to occur in CCFL.

Considering that the purpose of the study was to discuss a broad subject and raise initial arguments for a research topic still underexplored in the scientific literature, which is the list of ingredients as nutrition information in food labeling, the authors have chosen the methodology procedure that would best address the study objective.

## 4. Conclusions

The recommendations and standards set by the Codex Alimentarius are not mandatory; however, they determine issues related to free trade and are recognized by organizations such as the World Trade Organization and food regulatory bodies including the US Food and Drug Administration, the European Food Standards Agency, and the Brazilian Health Regulatory Agency. Thus, Codex Alimentarius recommendations are highly relevant and have a large impact on regulations and public policies worldwide. The fact that the list of ingredients is a compulsory component of food labels does not replace or nullify the relevance of discussing the list as a source of nutrition information. Nutrition labeling and food labeling are based on different principles. Therefore, food and nutrition labeling regulations are often separate, with different principles for application and surveillance. 

Regulatory discussions that consider the premises of nutrition and labeling as a tool for food choices are carried out within the scope of nutrition labeling. However, the development of regulations and public policies in this area are focused on nutrient contents. It is suggested that nutrition labeling be discussed as a tool for food choices in the context of public health from a broader, consistent, convergent perspective, considering not only nutrients but, above all, food composition (i.e., the ingredients that compose foods), so that consumers can make informed choices.

Additionally, it is noteworthy that in the public health area, there are several examples of guidance for consumers concerning nutrient adequacy for a healthy diet. However, there is a need for guidance and communication strategies for consumers to make a qualitative analysis of their diets, in order to effectively use the list of ingredients to make healthier choices. 

With this perspective, we recommend the inclusion of the list of ingredients as a nutrition labeling component in Codex Alimentarius Food Labeling Standard and Nutrition Labeling Guideline. This way, the ingredients, not just the nutrients, would be considered nutritional aspects of a food within the regulatory framework of food labeling around the world. Therefore, the analysis of the nutritional quality of a packaged food would be officially based on the ingredients that compose it, not only the nutrient profile. Moreover, this recommendation would be an opportunity to discuss improvements on the list of ingredients’ readability and comprehension by the consumers, such as the inclusion of QUID (quantitative declaration of ingredients), which was on the agenda of several meetings of the Codex Committee on Food Labelling and discussed as a tool to help consumers avoid misinterpretation of food labels and make more informed food choices.

## Data Availability

Not applicable.

## Supplementary Materials

The following supporting information can be downloaded at: https://www.mdpi.com/article/10.3390/nu15214513/s1, Supplementary Material S1: Scoping review protocol.
