# Supplementary material for: Is the List of Ingredients a Source of Nutrition and Health Information in Food Labeling? A Scoping Review
Source: Nutrients. 2023 Oct 25;15(21):4513. doi: 10.3390/nu15214513 (PMC10647387; doi:10.3390/nu15214513)
Supplement: Supplementary file 1 [file nutrients-15-04513-s001.zip › nutrients-2590780-supplementary.pdf]

nutrients MDPI

Supplementary Materials

Is the List of Ingredients a Source of Nutrition and Health Information in Food Labeling? A Scoping Review

Mariana V. S. Kraemer [1], Ana Carolina Fernandes [1], Maria Cecília C. Chaddad [2], Paula L. Uggioni [1], Greyce L. Bernardo [1] and Rossana P. C. Proença [1,]*

Joanna Briggs Institute (JBI) Scoping Review Protocol (Peters et al., 2020; Tricco et al., 2018)

Objective: Discuss the role of the list of ingredients as a source of nutrition and health information in food labeling.

Title: Is the list of ingredients a source of nutrition and health information in food labeling? A scoping review

Review question: Is the list of ingredients a source of nutrition and health information in food labeling?

PCC (Population, Concept, and Context) framework:

Population:	Food labeling of packaged foods
Concept:	List of ingredients
Context:	List of ingredients as nutrition and health information in food labeling

Inclusion criteria:

- All meeting reports of the Codex Committee on Food Labelling since its creation, in 1965, to the present day

Uniterms:

- "Ingredient"; “Ingredients”
- “List of ingredient”
- “Health”
- “Nutrition”

Sections of the manuscript:

Background and Objective

Methods

Results and discussion

The list of ingredients as discussed by the Codex Committee on Food Labelling: Historical milestones from a health and nutrition perspective

Timeline of major discussions on the role of the list of ingredients in food labeling. Has the list of ingredients ever been discussed and regarded as nutrition information? Have debates been held on the relationship between the list of ingredients (or any information contained therein) and health and nutrition issues? Has the list of ingredients been related to nutrition labeling?

Nutrition labeling: Do only nutrients matter for health?

- Nutrition labeling as a synonym of nutrient composition
- Overvaluation of nutrients in discussions on diet and health
- Examples of official documents and scientific discussions on the topic

Limitations

Conclusion
